# High expression of EZH2 as a marker for the differential diagnosis of malignant and benign myogenic tumors

**DOI:** 10.1038/s41598-018-30648-7

**Published:** 2018-08-17

**Authors:** Ning Zhang, Zhi Zeng, Shaobo Li, Fei Wang, Peng Huang

**Affiliations:** 1 School of Basic Medicine, Shanghai University of Medicine and Health Sciences, Shanghai, 201318 People’s Republic of China; 2 Department of Pathology, Renmin Hospital of Wuhan University, Wuhan, 430060 Hubei Provicne People’s Republic of China; 3 Pathology Center, Shanghai General Hospital/Faculty of Basic Medicine, School of Medicine, Shanghai Jiao Tong University, Shanghai, 200025 People’s Republic of China; 4 Second Department of Neurosurgery, The First Affiliated Hospital of Kunming Medical University, Kunming, 650032 Yunnan Province People’s Republic of China; 5 School of Clinical Medicine, Shanghai University of Medicine and Health Sciences, Shanghai, 201318 People’s Republic of China

## Abstract

Overlap in morphologic features between malignant and benign myogenic tumors, such as leiomyosarcoma (LMS) vs. leiomyoma as well as rhabdomyosarcoma (RMS) vs. rhabdomyoma, often makes differential diagnosis difficult and challenging. Here the expressions of Enhancer of Zeste Homolog 2 (EZH2), Suppressor of Zeste 12 (SUZ12), retinoblastoma protein associated protein 46 (RbAp46), Embryonic Ectoderm Development (EED) and ki-67 protein were detected by immunohistochemistry to evaluate their values in differential diagnosis. The expression of EZH2 mRNA was investigated by analyzing the Gene Expression Omnibus Datasets. The results demonstrated that EZH2 protein was detected in 81.25% (26/32) of LMS and 70.58% (36/51) of RMS, whereas none of leiomyoma (n = 16), rhabdomyoma (n = 15) and normal tissues (n = 31) showed positive immunostaining (*p* < 0.05). EZH2 protein was found to have a sensitivity of 91.30% and specificity of 100% in distinguishing well-differentiated LMS from cellular leiomyoma, and a sensitivity of 92.86% and specificity of 100% in distinguishing well-differentiated embryonal rhabdomyosarcoma (ERMS) from fetal rhabdomyoma. Besides, the expression of EZH2 mRNA was higher in LMS and RMS than in benign tumors (*p* < 0.05). The expressions of SUZ12 and RbAp46 protein were higher in RMS than in rhabdomyoma (*p* < 0.05). Conclusively, the high expression of EZH2 is a promising marker in distinguishing well–differentiated LMS from cellular leiomyoma, or well–differentiated ERMS from fetal rhabdomyoma, and the upregulation of EZH2 protein expression may occur at transcriptional level.

## Introduction

Overlap in morphologic features between malignant and benign myogenic tumors, such as well-differentiated leiomyosarcoma (LMS) vs. cellular leiomyoma as well as well-differentiated rhabdomyosarcoma (RMS) vs. fetal rhabdomyoma, often makes differential diagnosis difficult and challenging. LMS mainly occurs in the pelvic retroperitoneum/abdomen of adults, with hypercellularity, nuclear atypia and active mitosis. However, well-differentiated LMS, with no excessive mitotic activity and undue nuclear atypia, often shows histological features similar to cellular leiomyoma. Cellular leiomyoma often shows the morphology features of increased cellularity, large tumor size and active mitotic activity. RMS is one of the most common sarcomas with high recurrence rates in infants and children. Embryonal rhabdomyosarcoma (ERMS) is a subtype of RMS. The well-differentiated ERMS is sometimes indistinguishable from fetal rhabdomyoma for lacks of cytologic atypia and active mitosis. Fetal rhabdomyoma is rare and often considered to be a mimic of well-differentiated ERMS. Immunohistochemical markers used for clinical diagnoses, such as MAS, vimentin, calponin, SMA and desmin, are not effective in differentiating these malignant tumors from benign tumors. To address this issue, highly sensitive and specific new markers are needed for differential diagnosis.

Enhancer of Zeste Homolog 2 (*EZH2*) is a key subunit of polycomb repressive complex 2 (*PRC2*) and represses its target genes by catalyzing histone methylation through the coordinate action of three other components of PRC2, including Suppressor of Zeste 12 (*SUZ12*), Embryonic Ectoderm Development (*EED*) and retinoblastoma protein associated protein 46 (*RbAp46*). Previous studies have reported that high expression of *EZH2* was found in various kinds of cancers, which contributed to tumor progression and poor prognosis^1–9^. In recent years, upregulation of *EZH2* expression was also discovered in certain sarcomas, including Ewing sarcoma, RMS, synovial sarcoma, osteosarcoma and chondrosarcoma. Ewing sarcoma and RMS are common pediatric sarcomas with high malignance. Ramaglia^10^ found that EZH2 was expressed with a different degree in 60% of 17 patients with Ewing sarcoma or RMS and it was significantly higher in patients presenting lymph node and/or distant metastases. Moreover, overexpression of EZH2 was positively relative to lower probability of survival. EZH2 expression can be induced by the fusion gene of EWS/FLI1 via binding to its promoter in Ewing sarcoma *in vivo*^11^. It was also demonstrated that EZH2 was aberrantly overexpressed in RMS primary tumors and cell lines, and downregulation of it *in vitro* can lead to muscle-like differentiation of RMS cells^12–14^. Some studies exist on synovial sarcoma revealed that EZH2 expressed in 76% of patients^15^. EZH2 mRNA and protein were highly expressed in poorly differentiated subtype. Even in the monophasic and biphasic subtypes, patients with high EZH2 score was characterized by high proliferation rate, large tumor size, distant metastasis and poor outcomes^16^. EZH2 overexpression was also detected in osteosarcoma^17^ and high grade chondrosarcomas^18^, which was significantly associated with aggressive behavior and poor prognosis. However, the expression of *EZH2* in malignant myogenic tumors, such as LMS and RMS, remains unknown. Specifically, the difference of its expression in malignant and benign tumors has not been elucidated yet.

In this study, we detected the expression of *EZH2* and three other components of *PRC2* in 32 LMS, 51 RMS, 16 uterine leiomyoma, 15 rhabdomyoma as well as 31 normal tissues to evaluate the application value of *EZH2* in differential diagnosis. In addition, the association between *EZH2* expression and clinicopathological characteristics, and the correlation of EZH2 with ki-67 protein were assessed to identify the significance in these malignant tumors. We also investigated *EZH2* mRNA expression by analyzing the Gene Expression Omnibus (GEO) Datasets.

## Results

### The EZH2 protein was highly expressed in LMS

**Figure 1:**
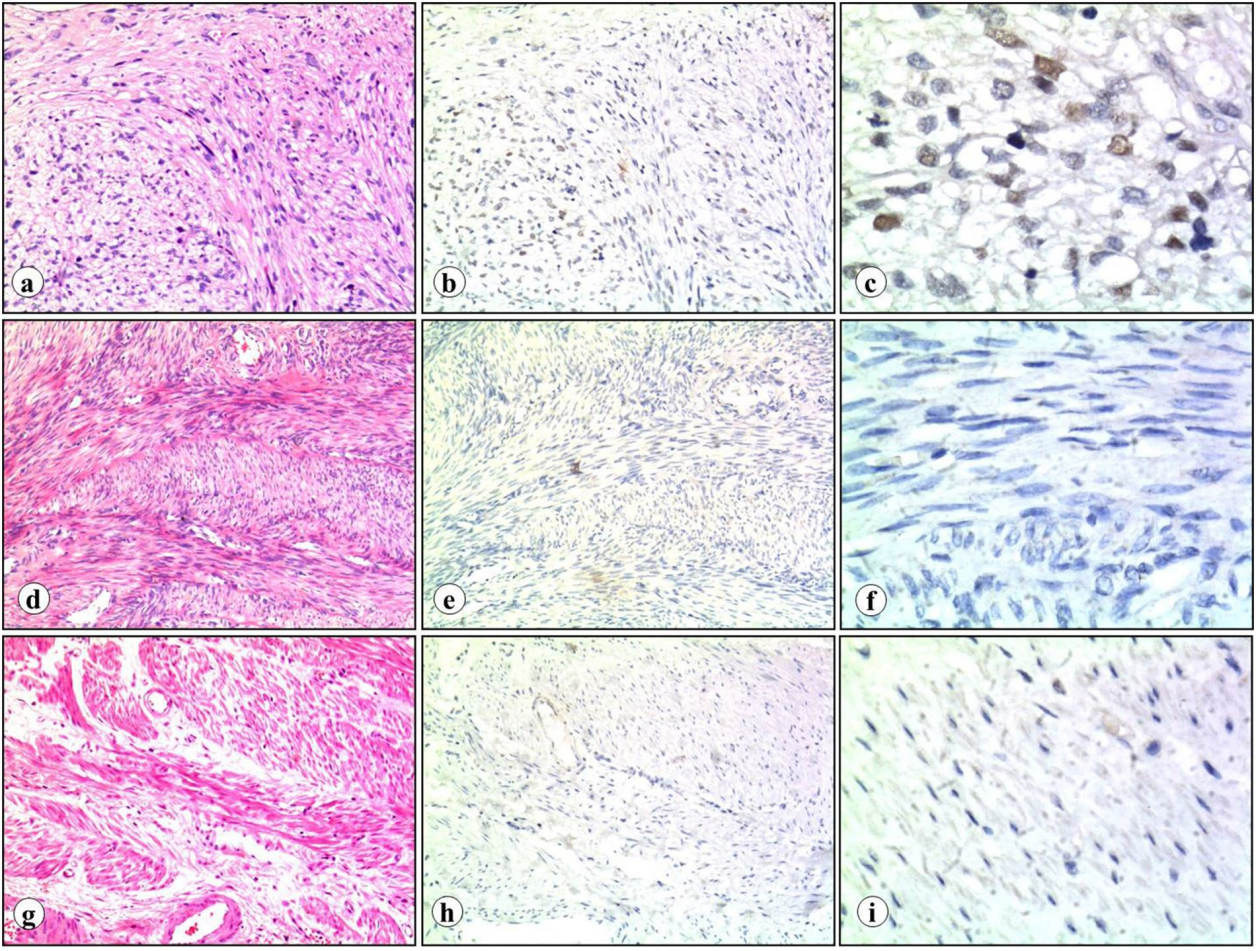
EZH2 expression in LMS, uterine leiomyoma and normal myometrium. LMS (**a**, H&E staining) showed positive staining for EZH2 protein (**b**,**c**). Brown color in nuclei indicates strong positive staining. In contrast, uterine leiomyoma (**d**, H&E staining) and normal myometrium (**g**, H&E staining) exhibited negative staining (**e**,**f**: uterine leiomyoma; **h**,**i**: normal myometrium) (**a**,**b**, **d**,**e** and **g**,**h**, original magnification x100; **c**,**f** and **i**, original magnification x400).

In this study, immunohistochemical analysis was performed in 64 specimens, including 32 LMS, 16 uterine leiomyoma and 16 normal myometrial to quantify and localize the expression of EZH2 protein. Microscopy pictures showed nuclear localization of EHZ2 in LMS (Fig. 1).

Totally, EZH2 protein expression was observed in 26 of 32 (81.25%) LMS, whereas none was found in uterine leiomyoma and normal myometrium (*p* < 0.05, Table 1). Of 32 LMS cases, 4 were scored 0 (negative staining), 2 were scored 1 (weak staining not exceeding 24% of tumor cells), 13 were scored 2 (weak staining between 25% to 49%), 16 were scored 2+  (moderate to strong staining exceeding 50%, Supplementary Table 1). Further subgroup analysis showed that EZH2 expression was found in 21 of 23 (91.30%) well-differentiated and significancently higher than that in cellular uterine leiomyoma (*p* < 0.05) (Table 2). According to the tumor sites, LMS were divided into two groups: uterine LMS and extra-uterine LMS. No significant difference in EZH2 protein expression level was found between the two groups (Table 3 and Supplementary Table 2).

**Table 1:** Summary of the expression of EZH2 protein in LMS, leiomyoma and myometrium.

Tumor type	n	EZH2 expression		PR (%)	*P* value
		+	−		
LMS	32	26	6	81.25	0.000*
Leiomyoma	16	0	16	0	
Myometrium	16	0	16	0	

LMS, leiomyosarcoma; *P < 0.05, by Chi-square analysis.

**Table 2:** Summary of the expression of EZH2 in well-differentiated LMS, cellular leiomyoma and myometrium

Group	n	EZH2 expression		PR (%)	*P* value
		+	−		
Well-differentiated LMS	23	21	2	91.30	0.000*
Cellular leiomyoma	16	0	16	0	
Myometrium	16	0	16	0	

LMS, leiomyosarcoma; *P < 0.05, by Chi-square analysis.

**Table 3:** Summary of the expression of EZH2 protein in uterine LMS and extra-uterine LMS

Tumor sites of LMS	n	EZH2 expression		PR (%)	*P* value
		+	−		
Uterus	14	12	2	85.71	0.672
Extra-uterus	18	14	4	77.78	

LMS, leiomyosarcoma; *P < 0.05, by Chi-square analysis.

### The EZH2 protein was highly expressed in RMS

**Figure 2:**
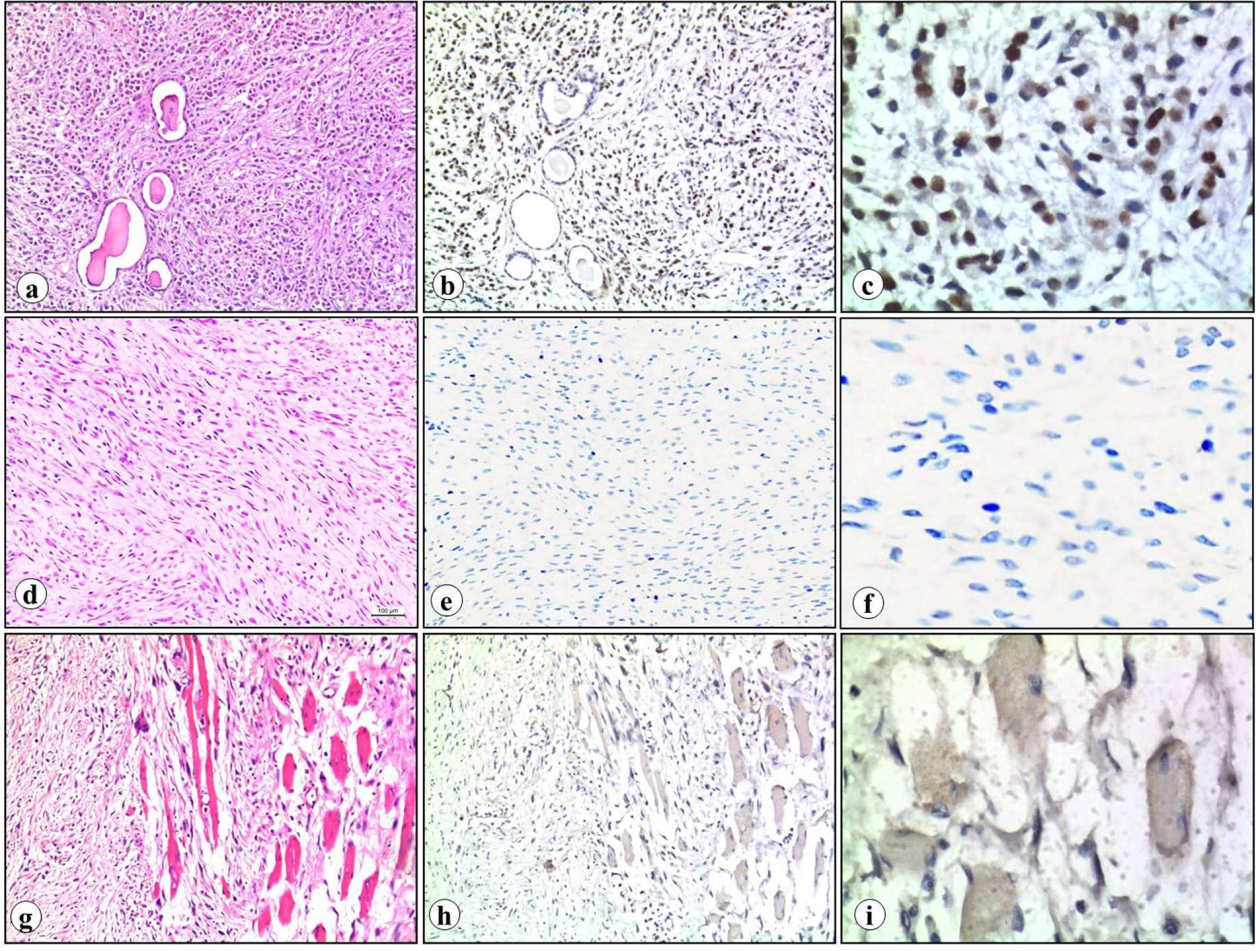
EZH2 expression in ERMS, fetal rhabdomyoma and TASM. ERMS (**a**, H&E staining) showed positive staining for EZH2 protein (**b**,**c**). Brown color in nuclei indicates strong positive staining. In contrast, fetal rhabdomyoma (**d**, H&E staining) and TASM (**g**, H&E staining) showed negative staining for EZH2 protein (**e**,**f**: fetal rhabdomyoma; h-i: TASM) (**a**,**b**, **d**,**e** and **g**,**h**, original magnification x100; c, f and i, original magnification x400, Scale bar,100 μm).

In this study, the expression of EZH2 protein was measured in 81 cases, 51 from RMS and 15 from fetal rhabdomyoma, as well as in 15 specimens of tumor adjacent skeletal muscle (TASM). Microscopy pictures showed nuclear localization of EHZ2 in RMS (Fig. 2). EZH2 protein expression was detected in 36 of 51 (70.58%) RMS, but not in rhabdomyoma and TASM (*p* < 0.05, Table 4). Of 51 RMS cases, 10 were scored 0 (negative staining), 5 were scored 1 (weak staining not exceeding 24%), 12 were scored 2 (weak staining between 25% to 49%), 24 were scored 2+ (moderate to strong staining exceeding 50%, Supplementary Table 3). Subgroup analysis indicated that EZH2 protein was found in 26 of 28 (92.86%) of well-differentiated ERMS and significantly higher than that in fetal rhabdomyoma (*p* < 0.05, Table 5).

**Table 4:** Summary of the expression of EZH2 in RMS, rhabdomyoma and TASM.

Tumor type	n	EZH2 expression		PR (%)	*P* value
		+	−		
RMS	51	36	15	70.58	0.000^*^
Rhabdomyoma	15	0	15	0	
TASM	15	0	15	0	

TASM, tumor adjacent skeletal muscle; *P < 0.05, by Chi-square analysis.

**Table 5:** Summary of the expression of EZH2 in embryonal RMS, rhabdomyoma and TASM

Tumor type	n	EZH2 expression		PR (%)	*P* value
		+	−		
Well-differentiated ERMS	28	26	2	92.86	0.000^*^
Fetal rhabdomyoma	15	0	15	0	
TASM	15	0	15	0	

ERMS, embryonal rhabdomyosarcoma; TASM, tumor adjacent skeletal muscle; *P < 0.05, by Chi-square analysis.

### High expression levels of EZH2 mRNA in LMS

**Figure 3:**
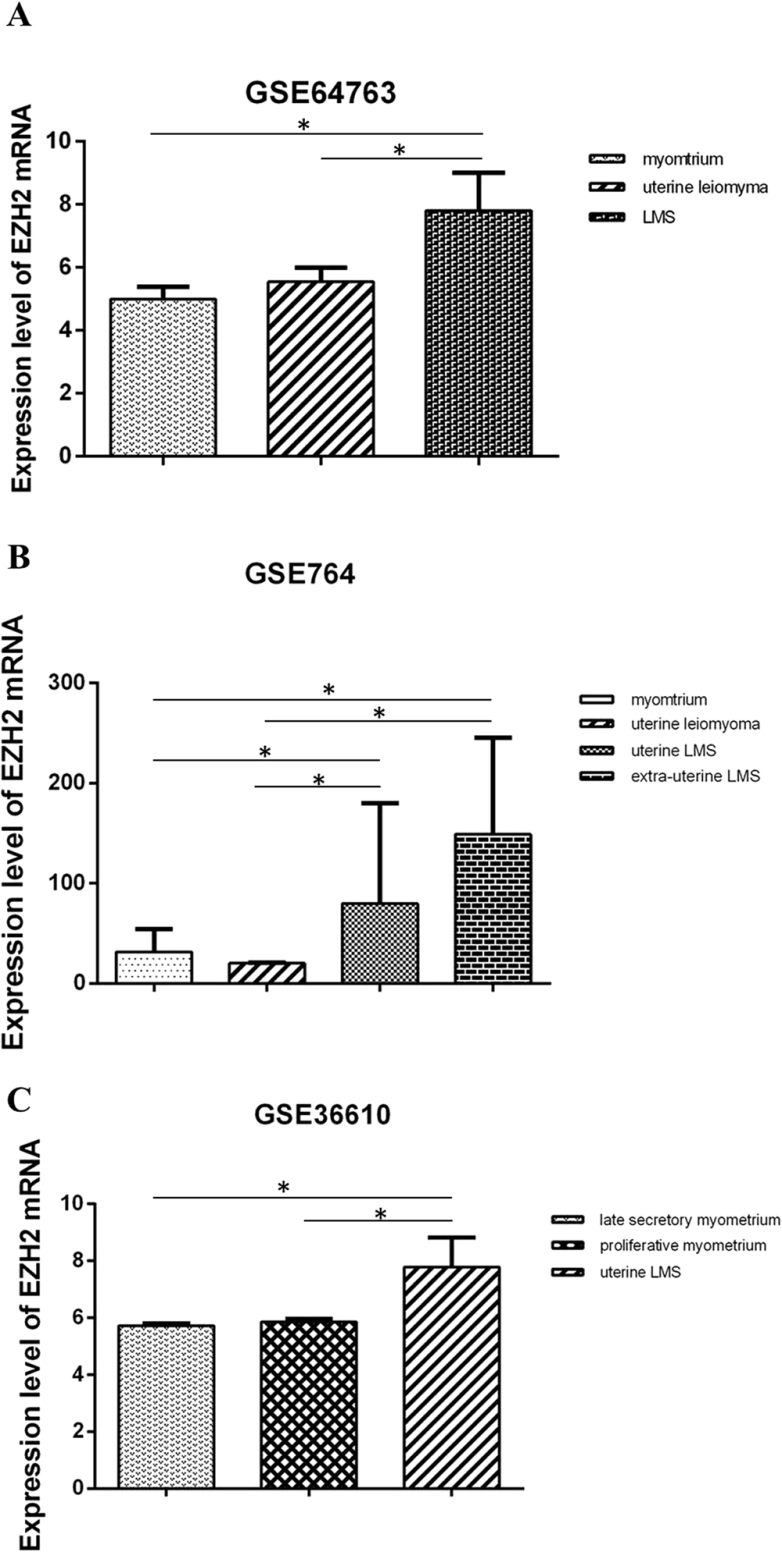
The expression of *EZH2* mRNA in human LMS, uterine leiomyoma and myometrium is detected by bioinformatics analysis in GEO datasets. **(A)** The data set from GSE64763 shows the relative expression level of *EZH2* mRNA in normal myometrium, uterine leiomyoma and LMS. **(B)** The data set from GSE764 shows the relative expression level of *EZH2* mRNA in myometrium, uterine leiomyoma, uterine LMS and extra-uterine LMS. **(C)** The data set from GSE36610 shows the expression level of *EZH2* mRNA in late secretory myometrium, proliferative myometrium and uterine LMS. (One-way ANOVA).

The data set GSE64763 (Fig. 3A) showed the relative expression levels of *EZH2* mRNA in LMS (n = 25), uterine leiomyoma (n = 25) and normal myometrium (n = 28) were 1.56, 1.11 and 1, respectively. *EZH2* mRNA expression in LMS were significantly higher than those in uterine leiomyoma and normal myometrium (p < 0.05).

The data set GSE764 (Fig. 3B) showed the relative expression levels of EZH2 mRNA in uterine LMS (n = 9), extra-uterine LMS (n = 4), uterine leiomyoma (n = 9) and normal myometrium (n = 4) were 2.54, 5.15, 0.64 and 1, respectively. The expression of *EZH2* mRNA in uterine and extra-uterine LMS were higher than those in uterine leiomyoma and normal myometrium (p < 0.05), respectively. And there was no difference in expression levels between uterine LMS and extra-uterine LMS.

As we known that the different stages of uterus depend on the changes of physiolgical characteristics of endometrium, not myometrium. In order to understand whether EZH2 mRNA expression in myometrium was affected by different statuses of endometrium, we investigated it in two phases of endometrium, including late secretory phase and proliferative phase. The data set GSE36610 (Fig. 3C) showed the relative expression levels of *EZH2* mRNA in uterine LMS (n = 12), myometrium which adjacent endometrium was at the stage of proliferation (n = 5) and myometrium which adjacent endometrium was at the stage of late secretory (n = 5) were 1.36, 1.02 and 1, respectively. It was significantly higher in EZH2 mRNA expression level in uterine LMS than that in the two latter (*p* < 0.05). No difference was found between the two different stages of endometrium.

### High expression levels of *EZH2* mRNA in RMS

**Figure 4:**
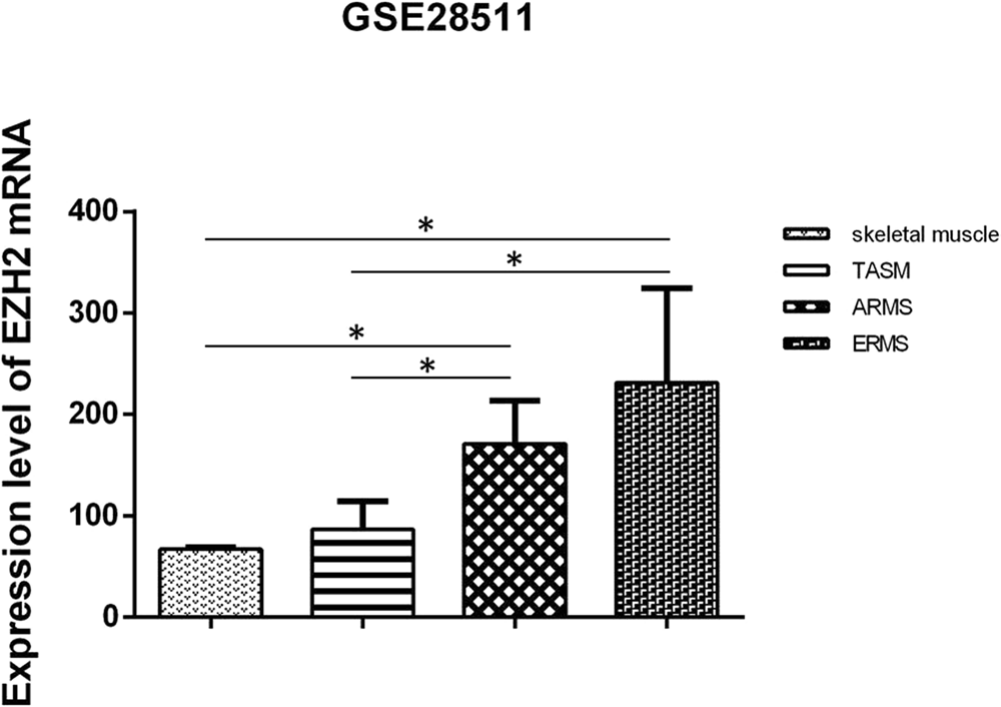
The data set from GSE28511 shows the relative expression level of *EZH2* mRNA in skeletal muscle, TASM, ARMS and ERMS. (One-way ANOVA).

The data set from GSE28511 was utilized to analyze the relative expression levels of *EZH2* mRNA in RMS, TASM and skeletal muscle. The result showed that the relative expression level of *EZH2* mRNA in ERMS (n = 8), alveolar rhabdmyosarcoma (ARMS) (n = 10), TASM (n = 3) and normal skeletal muscle (n = 3) were 3.44, 2.55, 1.28 and 1, respectively. *EZH2* mRNA expression in ERMS and ARMS were significantly higher than those in TASM and skeletal muscle, respectively (p < 0.05). And there were no significant differences in expression levels between ERMS and ARMS, or between TASM and skeletal muscle (Fig. 4).

### Expression levels of *EZH2* mRNA in other sarcomas at tissue and cellular levels

**Figure 5:**
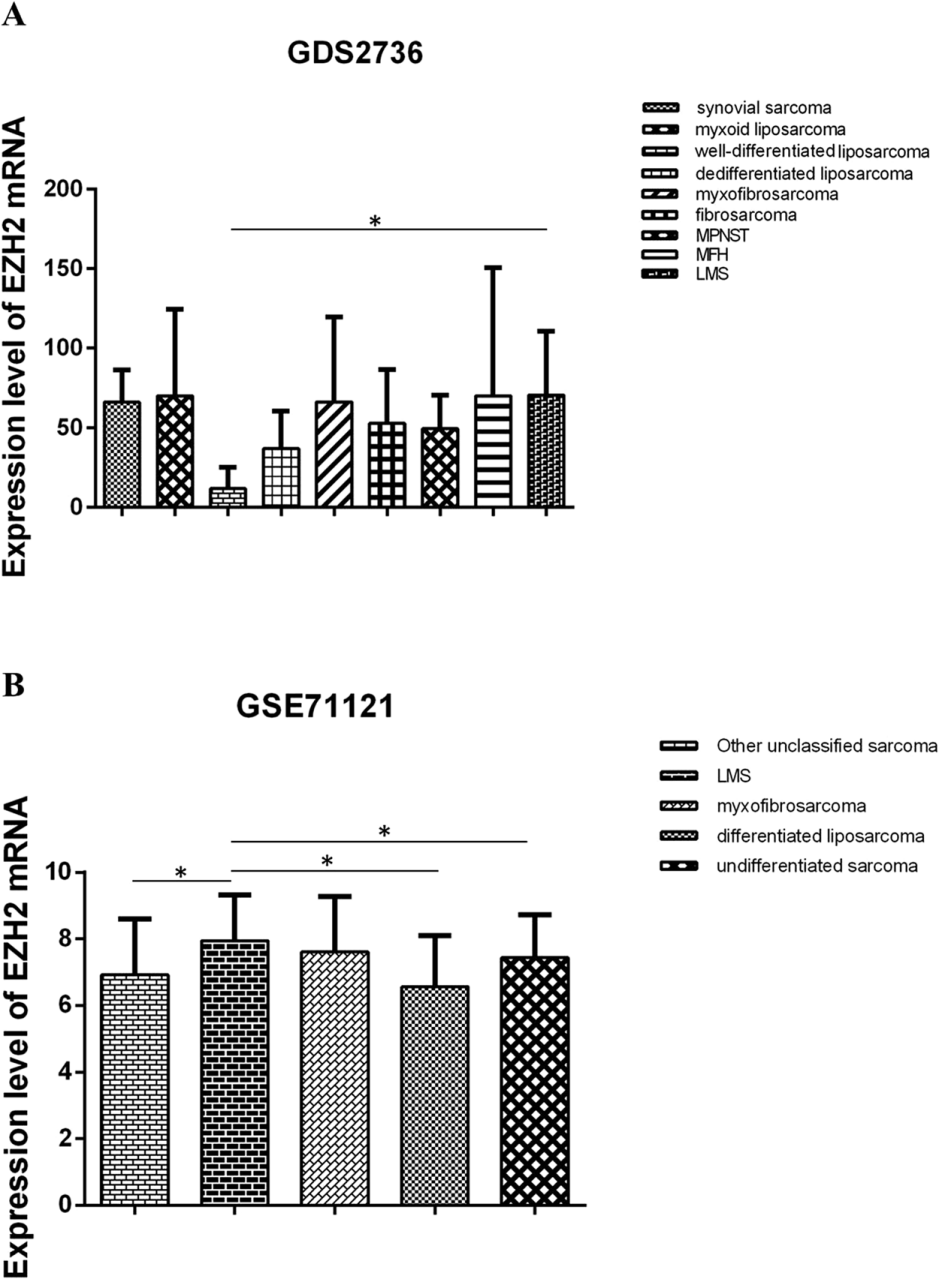
The expression of *EZH2* mRNA in LMS and other sarcomas at tissue level is detected by bioinformatics analysis in GEO datasets. **(A)** The data set from GDS2736 shows the expression level of *EZH2* mRNA in LMS and 8 kinds of other sarcomas. **(B)** The data set from GSE71121 shows the expression level of *EZH2* mRNA in LMS, myxofibrosarcoma, differenctiated liposarcoma and undifferentiated sarcoma and other types. (One-way ANOVA).

In order to know the difference in expression level of EZH2 mRNA between LMS and other sarcomas, we investigated EZH2 mRNA in 8 kinds of sarcoma tissues. The data set GDS2736 (Fig. 5A) showed the expression level of EZH2 mRNA in LMS (n = 6) was higher than that in well-differentiated liposarcoma (n = 3) (*p* < 0.05). And there was no significant difference in expression levels between LMS and synovial sarcoma (n = 16), myxoid liposarcoma (n = 19), dedifferentiated liposarcoma (n = 15), myxofibrosarcoma (n = 15), fibrosarcoma (n = 4), malignant peripheral nerve sheath tumor (MPNST, n = 3) and malignant fibrous histiocytoma (MFH, n = 21).The data set GSE71121 (Fig. 5B) revealed that the expression level of EZH2 mRNA in LMS (n = 90) was higher than that in differentiated liposarcoma (n = 44), undifferentiated sarcoma (n = 88) and other unclassified sarcoma (n = 45) (p < 0.05). And no significant difference was found between LMS and myxoid liposarcoma (n = 42).

**Figure 6:**
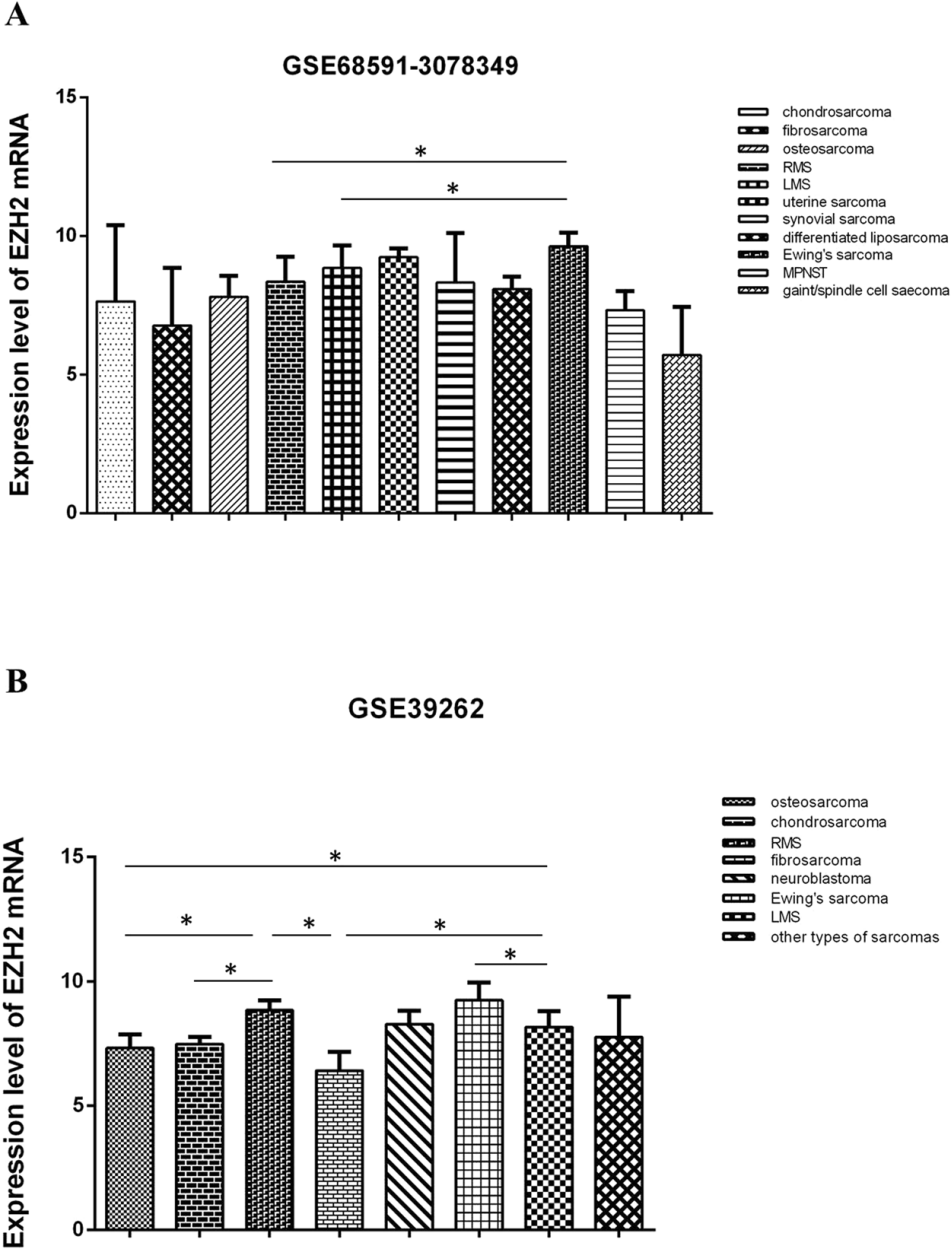
The expression of *EZH2* mRNA in LMS, RMS and other sarcomas at cellular level is detected by bioinformatics analysis in GEO datasets. **(A)** The data set from GSE68591-3078349 shows the expression level of *EZH2* mRNA in LMS, RMS and 10 kinds of cell lines of other sarcomas. **(B)** The data set from GSE39262 shows the expression level of *EZH2* mRNA in LMS, RMS and 12 kinds of cell lines of sarcomas.

Additionally, EZH2 mRNA in cell lines of other sarcomas was also investigated. The data set GSE68591-3078349 (Fig. 6A) showed the expression level of EZH2 mRNA in LMS was significantly lower than Ewing’s sarcoma (*p* < 0.05). And there was no significant difference in expression levels between LMS and chondrosarcoma, fibrosarcoma, osteosarcoma, uterine sarcoma, synovial sarcoma, differentiated liposarcoma, MPNST and giant/spindle cell sarcoma. It also indicated that the expression level of EZH2 mRNA in RMS was lower than Ewing’s sarcoma (*p* < 0.05). And no significantly difference was found between RMS and the above nine kinds of cell lines of sarcomas. The data set GSE39262 (Fig. 6B) showed that the expression level of EZH2 mRNA in LMS was higher than that in osteosarcoma, fibrosarcom and Ewing sarcoma (p < 0.05). And there was no significant difference in expression level between LMS and chondrosarcoma, neuroblastoma and other six kinds of sarcoma (liposarcoma, MFH, malignant primitive neuroectodermal tumor, desmoplastic round cell tumor, poorly differentiated liposarcoma cell line and synovial sarcoma). It also revealed that the expression level of EZH2 mRNA in RMS was higher than that in osteosarcoma, chondrosarcoma and fibrosarcoma (p < 0.05). No significant difference was found between RMS and chondrosarcoma, neuroblastoma and the above six sarcomas.

### The expression of EZH2 protein was highly sensitive and specific to LMS and RMS

Totally, EZH2 protein was found to have a sensitivity of 81.25% and specificity of 100% in distinguishing LMS from leiomyoma. Subgroup analysis showed the sensitivity and specificity of EZH2 protein were 91.30% and 100% in well-differentiated LMS. The accuracy is 96.36% in differentiating well-differentiated LMS from cellular leiomyoma.

The sensitively and specificity of RMS is 70.58% and 100% respectively. Further analysis revealed that EZH2 protein was found to have a sensitivity of 92.86% and specificity of 100% in well-differentiated ERMS. The accuracy of is 94.12% in differentiating well-differentiated ERMS from fetal rhabdomyoma (Table 6).

**Table 6:** The sensitivity, specificity and accuracy of EZH2 protein in well-differentiated LMS and ERMS.

Tumor type	EZH2 protein		
	Sensitivity (%)	Specificity (%)	Accuracy (%)
Well-differentiated LMS	91.30	100	96.36
Well-differentiated ERMS	92.86	100	94.12

LMS, leiomyosarcoma; ERMS, embryonal rhabdomyosarcoma.

### Association of EZH2 protein with clinicopathological features of LMSand RMS

The correlation between EZH2 protein expression in LMS and RMS and clinicopathological characteristics was analyzed (Table 7).

**Table 7:** The relationship between EZH2 expression and clinicopathological characteristics of LMS and RMS.

Clinicopathological characteristics	EZH2 expression			PR(%)	*P*
	n	+	−		
**LMS**					
**Gender**					
Males	15	12	3	80.00	1.000
Females	17	14	3	82.35	
**Age(years)**					
≤50	16	12	4	75.00	0.654
>50	16	14	2	87.50	
**Maximum diameter of tumor (cm)**					
≤8.5	16	11	5	68.75	0.172
>8.5	16	15	1	93.75	
**Tumor site**					
uterus	14	12	2	85.71	0.672
extra-uterus	18	14	4	77.78	
**ki-67**					
0–1^+^	18	15	3	83.33	1.000
2^+^-3^+^	14	11	3	78.57	
**RMS**					
**Gender**					
Males	26	18	8	69.23	1.000
Females	25	18	7	72.00	
**Age(years)**					
≤18	22	16	6	72.73	1.000
>18	29	20	9	68.97	
**Maximum diameter of tumor (cm)**					
≤3.0	30	23	7	76.67	0.351
>3.0	21	13	8	61.90	
**Lymphatic metastasis**					
Y	15	10	5	66.67	0.743
N	36	26	10	72.22	
**ki-67**					
0–1^+^	20	12	8	60.00	0.219
2^+^-3^+^	31	24	7	77.42	

^*^P < 0.05, by Chi-square analysis.

The age of patients with LMS ranged from 28 to 83 years, and the maximum diameter of tumors ranged from 3 to 15 cm. In 32 LMS cases, 14 (43.75%) patients developed the tumor in uterus and 18 (56.25%) in extra-uterus. 51 patients with RMS were also included in this study. The ages of patients ranged from 3 months to 66 years. As RMS usually occurs in infants and children, age of 18 was picked as an age cut off for RMS. The maximum diameter of the tumors ranged from 0.5 to 13 cm. No significant association was found between EZH2 expression and clinicopathological features of LMS and RMS, including gender, age, tumor size, tumors site and lymphatic metastasis.

In previous studies, EZH2 protein was highly expressed and significantly associated with overexpression of ki-67 protein in some solid tumors, such as breast cancer^19,20^ and gastric cancer^21,22^. To clarify the correlation between them in LMS and RMS, we further investigated the expression of ki-67 protein and no association was observed.

### The expression of SUZ12, EED and RbAp46 in LMS and RMS

EZH2 is a methyltransferase that acts in coordination with three other components of *PRC2*, including *SUZ12*, *RbAp46* and *EED*. In this study, we also investigated the expression of these proteins in LMS, RMS as well as benign tumors.

SUZ12 and EED protein were detected in 18 of 32 (56.25%) and 14 of 32 (43.75%) LMS, respectively, and they were lower than that in uterine leiomyoma (16/16,100%) and normal myometrium (16/16,100%) (p < 0.05). No difference of RbAp46 expression was found between LMS and leiomyoma.

SUZ12 and RbAp46 protein expression were observed in 37 of 51 (72.55%) and 35 of 51 (68.63%) RMS, respectively, and they were significantly higher than that in rhabdomyoma and TASM (p < 0.05). No significant difference in EED expression was found between RMS and rhabdomyoma (Table 8). The sensitivity and specificity of SUZ12, RbAp46 and EED in LMS and RMS was analyzed (Table 9).

**Table 8:** Summary of the expression of SUZ12, EED and RpAB46 in LMS and RMS.

Tumor type	n	SUZ12 expression		PR (%)	*P*	RbAp46 expression		PR (%)	*P*	EED expression		PR (%)	*P*
		**+**	**−**			**+**	**−**			**+**	**−**		
LMS	32	18	14	56.25	0.000*	2	30	6.25	0.242	14	18	43.75	0.000*
Leiomyoma	16	16	0	100		0	16	0		16	0	100	
Myometrium	16	16	0	100		0	16	0		16	0	100	
Rhabdomyosarcoma	51	37	14	72.55	0.000*	35	16	68.63	0.000*	38	13	74.51	0.226
Rhabdomyoma	15	2	13	13.33		4	11	26.66		12	3	80.00	
TASM	15	0	15	0		0	15	0		14	1	93.33	

^*^P < 0.05, by Chi-square analysis.

**Table 9:** The sensitivity and specificity of SUZ12, RpAB46 and EED in LMS and RMS.

	SUZ12	RbAp46	EED
**LMS**			
Sensitivity (%)	56.25	6.25	43.75
Specificity (%)	0	100	0
**RMS**			
Sensitivity (%)	72.55	68.63	74.51
Specificity (%)	93.33	86.67	13.33

## Discussion

Generally, the main pathological criteria for differentiating malignant tumors from benign tumors include cytologic atypia, mitotic figures and necrosis. However, overlapping pathological features are frequently seen between malignant and benign myogenic tumors, which often make differential diagnosis difficult and challenging.

Well-differentiated LMS is sometimes indistinguishable from cellular leiomyoma for lacking of hypercellularity, active mitosis and excessive cytologic atypia. However, cellular leiomyoma in deep soft tissue is easily misdiagnosed with well-differentiated LMS for the similar histopathological characteristics. The present study demonstrated the utility of EZH2 immunohistochemistry for differentiating well-differentiated LMS from cellular leiomyoma. EZH2 protein was found to have a sensitivity of 81.25% and specificity of 100% in distinguishing LMS from leiomyoma. Further subgroup analysis showed that the sensitivity and specificity of EZH2 in well-differentiated LMS were 91.30% and 100%, respectively, which indicated that high expression of EZH2 is highly sensitive and specific to LMS, especially to well-differentiated LMS. The transcriptional level analysis showed that the expression of *EZH2* mRNA in LMS is higher than that in leiomyoma and myometrium, which suggested that the upregulation of EZH2 expression in LMS may occur at the transcriptional level. RMS is a highly aggressive sarcoma with high recurrence rates in infants and children. ERMS, composed of primary-differentiated blastema cells and rhabdomyoblasts, often show similar pathological features to fetal rhabdomyoma. Fetal rhabdomyoma is rare and pathologists from basic hospitals do not have enough diagnostic experience, which may increase the risk of misdiagnosis. Under-treatment or over-treatment is unavoidable once misdiagnosis is made. In this study, EZH2 protein expression was detected in 70.58% of RMS, but none was found in rhabdomyoma and TASM. The subtype analysis showed the sensitivity and specificity of EZH2 protein in ERMS were 89.47% and 100%, respectively, which illustrated that EZH2 expression is highly sensitive and specific to ERMS and it can be utilized as a reliable marker for differentiating ERMS from rhabdomyoma. The transcriptional level analysis indicated that *EZH2* mRNA expression in RMS was higher than that in TASM and skeletal muscle.

The mechanisms of high EZH2 protein expression in malignant tumors are mainly due to the overexpression of the oncogenes and down-regulation of the suppressors of *EZH2*. *RAS/KARS* mutation promotes the expression of *EZH2* by activating the *MEK-ERK-ELK1* and *PI3K-AKT* signaling pathways of pancreatic cancer and non-small cell lung cancer^23,24^. The *Rb-E2F* signaling pathway up-regulates *EZH2* expression by binding to the promoter of *EZH2* in bladder cancer and small cell lung cancer^25,26^. Similarly, the fusion gene EWS/Friend leukemia integration 1 transcription factor (FLI1) induced EZH2 expression by directly binding to its promotor in Ewing’s sarcoma cell lines^11^.When the expression levels of micro-RNAs, such as miRs-25, -26a, -101, -138 and -214, were decreased, the expression of *EZH2* was increased by interacting directly with the 3′-UTR of *EZH2* in malignant tumors^27–30^.

The associations between EZH2 expression and the clinicopathological features of LMS and RMS were analyzed. Contrary to previous studies^19–22^, we found no correlation between EZH2 and ki-67 proteins either in LMS or RMS. We do not know what the mechanism is, and it perhaps lacks the reaction sites in the signal pathway.

EZH2 can’t play the role as a methyltransferase without the synergistic action of *SUZ12*, *RbAp46* and *EED*. Thus, we further investigated the expression of those three proteins both in malignant and benign tumors. SUZ12 and EED protein expression in LMS was deceased comparing with leiomyoma and myometrium. Recent studies reported that mutations of *SUZ12* and *EED* had occurred in malignant peripheral nerve sheath tumors as well as head and neck high-grade malignant peripheral nerve sheath tumors had led to the loss of trimethylation at lysine 27 of histone H3 (*H3K27me3*), a downstream gene of *EZH2*^31,32^. We plan to explore the mechanism of down-regulation of the expression of *SUZ12* and *EED* in LMS. Moreover, the expression of SUZ12 and RbAp46 in RMS was higher than that in rhabdomyoma and TASM. The sensitivity of SUZ12 and RbAp46 in ERMS was 72.55% and 68.63%, respectively, and the specificity was 93.33% and 86.67% respectively, which illustrated that SUZ12 and RbAp46 were highly specific to RMS and they may become supplementary indicators together with *EZH2* for differentiating RMS from rhabdomyoma.

In conclusion, EZH2 was highly expressed in LMS and RMS and it may be utilized as a new marker for differentiating well–differentiated LMS from cellular leiomyoma, or ERMS from rhabdomyoma. In addition, the upregulation of EZH2 protein expression in LMS and RMS may occur at transcriptional level. SUZ12 and RbAp46 may be used as supplementary indicators together with EZH2 for differentiating RMS from rhabdomyoma.

## Materials and Methods

### Patients and tissue samples

A total of 145 cases were collected from the Department of Pathology of Shanghai University of Medicine and Health Science. 32 LMS, 16 cellular and mitotically active uterine leiomyoma, 16 smooth muscle of myometrial, 51 RMS, 15 fetal rhabdomyoma (extracardiac) and 15 tumor-adjacent skeletal muscle (TASM) were selected and identified. Among 32 LMS cases, sites of involvement are as following: 6 were located in the retroperitoneum, 6 in the lower extremity, 2 in the orbit, 4 in the arteries, and 14 in the uterus. According to the degree of differentiation, LMS were divided into two subgroups: well-differentiated LMS (n = 23) and moderately-poorly differentiated LMS (n = 9). And the former were selected to compare with cellular leiomyoma at expression level of EZH2 protein.

Two histopathologic subtypes of RMS, including ERMS (n = 38) and ARMS (n = 13) were involved in this experiment. In group of ERMS, 28 cases of well-differentiated, 6 cases of moderately-differentiated and 4 cases of poorly-differentiated ERMS were included and the case of well-differentiated ERMS were selected to compare with fetal rhabdomyoma at the expression level of EZH2 protein. 12 cases of myxoid variant and 3 cases of intermediate form were included in the group of fetal rhabdomyoma.

Patients had given their written informed consent. Clinical information was collected from medical records. Then, the tissue microarrays, composed of core areas of 1.5-mm diameters taken from paraffin-embedded tissues using a Quick Ray Manual Tissue Microarrayer (UNITMA, Korea), were prepared by Wuhan Iwill Biological Technology Co., Ltd. The study was approved by the Ethics Committee of Shanghai University of Medicine and Health Sciences and experiments were performed in accordance with the relevant guideline and regulation of this committee.

### Immunohistochemistry

Tissue microarrays of 4-μm thickness were deparaffinized, hydrated and rinsed in PBS. After antigen retrieval by exposure to microwaves for 10 minutes, peroxide blocking was performed with 3% H_2_O_2_ at room temperature for 30 minutes. The sections were blocked with goat serum and incubated with EZH2 antibody (1:200; catalog number: 5246, Cell Signaling Technology, MA, U.S.), SUZ12 antibody (1:100; catalog number: ab12073, Abcam, Cambridge, UK), RbAp46 antibody (1 µg/ml; catalog number: ab3535, Abcam, Cambridge,UK), EED antibody (1:200; catalog number: ab96801, Abcam, Cambridge, UK) and ki-67 antibody (1:400, catalog number: 12202, Cell Signaling Technology, MA, U.S.) at 4 °C overnight. Then, the samples were probed with biotinylated secondary antibody and high-sensitivity HRP-conjugated streptavidin (kit catalog number: 9710, Fuzhou Maixin Biotech, China) at room temperature for 20 minutes each. All slides were stained with 3, 30-diaminobenzidine and counterstained with hematoxylin. Colorectal cancer tissue was used as a positive control. A negative control for antibodies was carried out by replacing the primary antibody with PBS.

### Evaluation of staining

The immunostained sections were evaluated by two independent pathologists and the expression of EZH2, SUZ12, EED, RbAp46 and ki-67 protein of each sample was scored according to the percentage of tumor cells with positive nuclear immunostaining and staining intensity. The score of each sample was the average score from the two observers’ evaluations. The percentage of positive tumor cells was scored as 0–4 (0 ≤ 10%, 1 = 10–24%, 2 = 25–49%, 3 = 50–74%, and 4 ≥ 75%), and the intensity of positive staining was quantified as 0–3(0 = negative, 1 = weak, 2 = moderate, and 3 = strong). The final score was obtained by multiplying these two scores. For statistical purposes, scores less than 2 were regarded as negative, and scores equal or more than 2 were considered to be positive.

### GEO database analysis

The mRNA data sets of LMS, RMS, other sarcomas as well as benign myogenic tumors and normal controls were downloaded from Gene Expression Omnibus (GEO) Datasets (https://www.ncbi.nlm.nih.gov/gds/), which is a publicly available gene expression database. Three independent data sets from GSE64763, GSE764 and GSE36610 were utilized to analyze the relative expression levels of *EZH2* mRNA in LMS, uterine leiomyoma and myometrium. One independent data set from GSE28511 was used to analyze the relative expression level of *EZH2* mRNA inRMS, TASM and skeletal muscle. Two independent data sets from GDS2736 and GSE71121 were utilized to analyze the difference in expression level of EZH2 mRNA between LMS and other sarcomas in tissue level. And two data sets from GSE68591-3078349 and GSE39262 were used to analyze the difference in expression level of EZH2 mRNA between LMS, RMS and other sarcomas in cellular level.

### Statistical analysis

All experimental data were analyzed by SPSS 19.0 statistical software (SPSS, Inc., Chicago, IL, USA). The expression difference of *EZH2*, *SUZ12*, *RbAp46* and *EED* between malignant tumors and benign tumors, as well as the association between EZH2 expression and clinicopathological parameters, were investigated by Chi-square tests or Fisher’s exact tests. The analysis of expression of *EZH2* mRNA in GEO datasets was performed by Student’s two tailed *t* test or one-way ANOVA. P < 0.05 was considered to be statistically significant for the analyses performed.

## Electronic supplementary material

Supplementary Dataset 1-3
