# Supplementary material for: High expression of EZH2 as a marker for the differential diagnosis of malignant and benign myogenic tumors
Source: Sci Rep. 2018 Aug 17;8:12331. doi: 10.1038/s41598-018-30648-7 (PMC6098067; doi:10.1038/s41598-018-30648-7)
Supplement: Supplementary file 1 — Supplementary Dataset 1-3 [file 41598_2018_30648_MOESM1_ESM.docx]

**Title page**

**Title:**

High expression of EZH2 as a marker for the differential diagnosis of malignant and benign myogenic tumors

**Author list:**

Ning Zhang^1*^, Zhi Zeng^2 *^, Shaobo Li^3^, Fei Wang^4^, Peng Huang^5#^

1. School of Basic Medicine, Shanghai University of Medicine and Health Sciences, Shanghai 201318, People's Republic of China
2. Department of Pathology, Renmin Hospital of Wuhan University, Wuhan 430060, Hubei Provicne, People's Republic of China
3. Pathology Center, Shanghai General Hospital/Faculty of Basic Medicine, School of Medicine, Shanghai Jiao Tong University, Shanghai 200025, People's Republic of China
4. Second Department of Neurosurgery, The First Affiliated Hospital of Kunming Medical University, Kunming 650032, Yunnan Province, People's Republic of China
5. School of Clinical Medicine, Shanghai University of Medicine and Health Sciences, Shanghai 201318, People's Republic of China

* These authors contributed equally to this work.

# Correspondence:

Peng Huang, School of Clinical Medicine, Shanghai University of Medicine and Health Sciences, No.279, ZhouZhu Road, Shanghai 201318, People's Republic of China.

Fax number/ telephone number: +86-21-65887292

Email: huangp_15@sumhs.edu.c

Supplementary table 1 Comparison of the distribution and intensity of EZH2 protein expression between LMS, leiomyoma and myometrium

| Group | n | Distribution of EZH2 expression | | | | |  | Intensity of EZH2 expression | | | |
| --- | --- | --- | --- | --- | --- | --- | --- | --- | --- | --- | --- |
|  |  | ≤10 | 10-24 | 25-49 | 50-74 | ≥75 |  | 0 | 1 | 2 | 3 |
| LMS | 32 | 4 | 2 | 13 | 10 | 3 |  | 4 | 15 | 10 | 3 |
| leiomyoma | 16 | 12 | 4 | 0 | 0 | 0 |  | 10 | 6 | 0 | 0 |
| myometrium | 16 | 14 | 2 | 0 | 0 | 0 |  | 12 | 4 | 0 | 0 |

LMS, leiomyosarcoma

Supplementary table 2 Comparison of the distribution and intensity of EZH2 protein expression between uterine and extra-uterine LMS

| Tumor sites  of LMS | n | Distribution of EZH2 expression | | | | | *p* | Intensity of EZH2 expression | | | | *p* |
| --- | --- | --- | --- | --- | --- | --- | --- | --- | --- | --- | --- | --- |
|  |  | ≤10 | 10-24 | 25-49 | 50-74 | ≥75 |  | 0 | 1 | 2 | 3 |  |
| Uterus | 14 | 2 | 1 | 5 | 5 | 2 | 0.923 | 1 | 6 | 6 | 2 | 0.519 |
| Extra-uterus | 18 | 2 | 1 | 8 | 5 | 1 |  | 3 | 9 | 4 | 1 |  |

LMS, leiomyosarcoma; *P<0.05, by Chi-square analysis

Supplementary table 3 Comparison of the distribution and intensity of EZH2 protein expression between RMS, rhabdomyoma and TASM

| Group | n | Distribution of EZH2 expression | | | | |  | Intensity of EZH2 expression | | | |
| --- | --- | --- | --- | --- | --- | --- | --- | --- | --- | --- | --- |
|  |  | ≤10 | 10-24 | 25-49 | 50-74 | ≥75 |  | 0 | 1 | 2 | 3 |
| RMS | 51 | 10 | 5 | 12 | 18 | 6 |  | 10 | 17 | 16 | 8 |
| Rhabdomyoma | 15 | 11 | 4 | 0 | 0 | 0 |  | 9 | 6 | 0 | 0 |
| TASM | 15 | 13 | 2 | 0 | 0 | 0 |  | 11 | 4 | 0 | 0 |

RMS, rhabdpmyosarcoma
